# Intra-Individual Relationship between Heart Rate Variability and the Underlying Heart Rate in Children and Adolescents

**DOI:** 10.3390/jcm13102897

**Published:** 2024-05-14

**Authors:** Martina Šišáková, Kateřina Helánová, Katerina Hnatkova, Irena Andršová, Tomáš Novotný, Marek Malik

**Affiliations:** 1 Department of Internal Medicine and Cardiology, University Hospital Brno, Jihlavská 20, 625 00 Brno, Czech Republic; sisakova.martina@fnbrno.cz (M.Š.); andrsova.irena@fnbrno.cz (I.A.);; 2 Department of Internal Medicine and Cardiology, Faculty of Medicine, Masaryk University, 62 500 Brno, Czech Republic; 3 National Heart and Lung Institute, Imperial College, London SW3 6LY, UK; k.hnatkova@imperial.ac.uk (K.H.); marek.malik@imperial.ac.uk (M.M.)

**Keywords:** paediatric population, heart rate variability, heart rate, regression slope, age, sympatho-vagal balance

## Abstract

**Background/Objective**: The relationship between heart rate and heart rate variability (HRV) indices has been repeatedly studied in adults but limited data are available on the relationship in paediatric populations. **Methods**: Continuous 12-lead electrocardiograms were recorded in 1016 healthy children and adolescents (534 females) aged 4 to 19 years during postural manoeuvres with rapid changes between 10-min positions of supine → sitting → standing → supine → standing → sitting → supine. In each position, the averaged RR interval was measured together with four HRV indices, namely the SDNN, RMSSD, quasi-normalised high-frequency components (qnHF), and the proportions of low- and high-frequency components (LF/HF). In each subject, the slope of the linear regression between the repeated HRV measurements and the corresponding RR interval averages was calculated. **Results**: The intra-subject regression slopes, including their confidence intervals, were related to the age and sex of the subjects. The SDNN/RR, RMSSD/RR, and qnHF/RR slopes were significantly steeper (*p* < 0.001) and the (LF/HF)/RR slopes were significantly shallower (*p* < 0.001) in younger children compared to older children and adolescents. **Conclusions**: The study suggests that sympathetic and vagal influences on heart rate are present in both younger and older children. With advancing age, the sympatho-vagal balance gradually develops and allows the vagal control to suppress the sympathetic drive towards higher heart rates seen in younger age children.

## 1. Introduction

Over recent decades, heart rate variability (HRV) has been the topic of numerous research studies and scientific publications. Indeed, the ESC/NASPE guideline document for HRV technology, physiological meaning, and possible practical implications [1] has been, since its publication in 1996, cited more than twenty-thousand times. Other standardisation documents [2,3] have received similar attention. As is well known, HRV studies include two major areas, namely the prediction of risk in cardiac patients [4,5,6] and the assessment of autonomic neuropathies [7,8,9]. Nevertheless, these two areas are far from exhaustive. Reports of many other possible applications of HRV investigations have been published. These include, among others, assessments of the progression of heart failure [10,11,12], of the risk in patients with epilepsy [13,14] and of the status of critically ill patients [15,16], evaluation of psychological stress [17,18] and of sleep pattern disturbances [19], monitoring of athletic training [20], detection of physical exhaustion [21], and many other topics. Complementing the “standard” time- and frequency-domain methods for HRV measurement, a variety of different additional techniques have also been proposed and shown to improve HRV values in potential applications [3,5,22,23,24].

Considering all the accumulated physiological knowledge, the technical capabilities of contemporary electrocardiography, and the available research experience, it seems surprising that HRV assessment does not belong to the standard diagnostic and disease assessment tools that are regularly employed in clinical practice, and that only specialised centres utilise the technology beyond research projects. This disparity between scientific knowledge and clinical applicability has been repeatedly discussed [25,26] and several potential reasons have been identified. Among others, the potential of HRV measurements for guiding therapeutic interventions has never been successfully tested in prospective randomised trials. The physiological interpretation of HRV measurements also depends on the circumstances under which the analysed heart rhythm data are recorded [27]. Without considering such circumstances, it is impossible to provide normal values for different HRV assessment techniques.

One of the problems with HRV data interpretation is the relationship to underlying heart rate. It is well known that at faster heart rates, the overall variability of cardiac periods decreases for both technical and physiological reasons. This HRV/heart rate relationship has been repeatedly described [10,27,28,29,30,31] and the practical meaning of the relationship has also led to occasional interpretation disputes [32,33,34]. Several attempts have also been made to develop a correction formula which would eliminate the effect of heart rate on the HRV values obtained by different measurement methods [35,36,37,38,39,40,41].

The relationship between HRV and the underlying heart rate in children is of particular interest [42,43] because young children are known to have higher resting heart rates. Previous investigations studied the relationship in population data, i.e., when merging heart rate and HRV measurements in different subjects into a group dataset in which the relationship might be investigated.

To complement these analyses, we report here a paediatric project which allowed us to study the HRV/heart rate relationship in each individual separately and to relate such intra-subject results to the sex and age of healthy children and adolescents. When designing the project, we did not have any “correction” of HRV measurements for the underlying heart rate in mind. Rather, we aimed at finding out whether the HRV/heart rate relationship shows any age-dependency when the data measurements are performed for different physiological conditions.

## 2. Materials and Methods

### 2.1. Study Population

The study investigated healthy school-aged children. Participants enrolment was conducted at schools in southern Silesia and Moravia. The protocol of the project was advertised at different pre-university education institutions (ranging between preparatory schools to pre-university establishments). To facilitate enrolment, the call for study enrolment offered a physical examination and clinical electrocardiogram (ECG) assessment. The study protocol was approved by the Ethics Committee of the University Hospital Brno on 13 June 2018 without any specific approval reference number. Written informed consent was obtained from the parents or legal guardians of the participants, unless the participants were of legal capacity age according to the laws of the Czech Republic, in which case they provided written informed consent personally. All aspects of the study adhered to the requirements of the Helsinki declaration. Standard demographic data were recorded, and medical histories were obtained from all participants.

### 2.2. Postural Provocations and ECG Recordings

Continuous 12-lead ECG recorders (SEER MC version 2, sampled at 1000 Hz, GE Healthcare, Wisconsin, WI, USA), with electrodes in the Mason–Likar position, were used. Each participant had a continuous ECG recorded during 70 min provocative postural manoeuvring protocol which consisted of six postural changes between seven body positions. Specifically, body positions were adopted in the following defined order: supine, sitting, standing, supine, standing, sitting, and supine. Each position was maintained for 10 min and, as per the study protocol, each of the position changes was accomplished within 20 s. No back or other additional support was provided during the sitting and standing positions.

All study investigations were performed in the mid-morning hours. Groups of up to 20 subjects of similar ages performed the positional changes at the same time. To facilitate adherence to the study protocol, younger children listened to non-exciting age-appropriate fairy stories. All other participants were investigated in a quiet, noise-free environment. No verbal or non-verbal contacts between the investigated subjects were allowed during the procedures. On the study days, none of the participants smoked or consumed caffeinated or alcoholic drinks before or during the investigation. During the morning hours preceding the study recordings, the participants followed standard education-related activities which did not include any physical exercise or other physically demanding tasks.

For feasibility and practical reasons, the ECG recorders were attached to the subjects and recordings started well before the provocative manoeuvres and terminated after the investigations. The duration of the ECG recordings before and after the provocative manoeuvring might have therefore differed between different subjects. Nevertheless, the exact start and stop times of each recording were documented in each case. This allowed us to delineate episodes corresponding to different postural positions and investigation phases within each continuous ECG.

### 2.3. QRS Detection

For the purposes of the computerised analysis of ECG signals, individual continuous recordings were divided into 10 s digital 12-lead portions with 5 s differences between the beginnings of subsequent positions. This led to a 5 s overlap between neighbouring portions. In each 10 s portion, QRS complexes were localised by four different algorithms, which were based on previously published QRS detection methods [44,45,46,47,48]. In ECG portions for which these different methods provided mutually consistent QRS positions, the results of the algorithms were accepted for further analyses. In cases of inconsistent results, the ECG portions were reviewed visually and, when necessary, the computerised QRS detections were manually corrected. The operators performing these steps of visual/manual review/correction were kept blinded in respect of the demographics of the recorded subject as well as of the timing of the reviewed 10 s ECG portions.

### 2.4. Heart Rate and Heart Rate Variability

The detection of the QRS positions in ECG portions provided a series of RR intervals for each postural position in each study subject. Changes between different postural positions led to heart rate instability and had to be consequently eliminated for the purposes of the HRV data analyses. Therefore, in each postural position, central 8 min intervals were considered (i.e., the first and last minutes of each postural position were eliminated from the analyses). Within the RR interval data corresponding to the central 8 min of each position, all consecutive 5 min sub-sections were considered if they did not contain any ectopic beats. If no 5 min sub-sections without ectopic beats were found, the postural position of the given subject was not considered in the analysis. Otherwise, the 5 min ectopic-free sub-section was identified that showed the least heart rate trending, i.e., was most free of any heart rate acceleration or deceleration. For this purpose, the slope of the linear regression between RR interval durations and the central time moments of the RR intervals was computed for each 5 min ectopic-free sub-section. The 5 min sub-section with the smallest absolute value of the slope was subsequently used to represent the given postural position.

These selected 5 min sub-sections provided the average values of RR interval durations, which were also converted into heart rate expressed in beats per minute (bpm).

Two time-domain HRV indices [1] were obtained from the RR sequences of the selected 5 min sub-sections:

- SDNN—standard deviation of all normal-to-normal RR intervals (note that the exclusion of ectopic beats meant that all the RR intervals were between QRS complexes classified as normal sinus rhythm beats), and
- RMSSD—root mean square of the differences between successive RR intervals.

Subsequently, discrete RR interval sequences of the selected 5 min sub-sections were subjected to cubic spline interpolation and resampled at 1000 Hz. These continuous RR interval signals were processed by Blackman–Tukey modification of Fast Fourier transformation with Hann window to obtain spectral HRV characteristics [1] of low-frequency components (LF—that is, the frequency power within the spectral band of 0.04–0.15 Hz) and high-frequency components (HF—that is, the frequency power within the spectral band of 0.15–0.40 Hz). The same technology has previously been used in a similar HRV study of adult subjects [49]. From the values of the HF and LF components, two spectral-domain HRV indices were obtained:

- LF/HF ratio—obtained as a proportion of the absolute values of the LF and HF components, and
- Quasi-normalised HF components, expressed as qnHF = HF/(LF + HF).

The quasi-normalised HF components approximated the standard normalised HF components that are normally derived from autoregressive spectral analysis [1,50], which was not used because it is not clear whether the same setting of autoregressive models would be appropriate for data from differently aged children. Because the quasi-normalised components were used, only the qnHF were calculated since qnLF = LF/(LF + HF) = 1 − qnHF. The qnHF values were expressed as percentages.

Both the LF/HF ratio and the qnHF components are derived from the LF and HF components, which leads to their non-linear reciprocal relationship, e.g., qnHF = 1/(LF/HF + 1). The reciprocal relationship is the basis for their interpretation. Increases in LF/HF and in qnHF approximate augmentations in sympathetic and vagal modulations, respectively.

Intentionally, we only considered the proportions of the HF and LF components rather than their numerical values separately. In recordings obtained under different conditions, such as the different postural positions used in this study, a physiological interpretation of spectral HRV components is only possible if both the HF and LF components are considered together (e.g., in the derived indices that we have just described) because of their numerical dependence on the total RR interval variance.

### 2.5. HRV/Heart Rate Relationship

The different postural positions in each subject provided, by study protocol, seven different values of heart rate and of corresponding HRV measurements. Thus, to express the intra-subject HRV/heart rate relationship, linear regressions were calculated for each study subject separately relating the measured HRV values to the averaged RR interval duration and to the heart rate.

Subsequently, for each HRV measure, the intra-subject regression residuals were computed for both types of regression. For each HRV measure, the type of linear regression (i.e., to RR average or to heart rate) was selected that, in the complete study population, led to lower regression residuals.

For the selected type of linear regression, double sided 90% confidence intervals of the intra-subject slopes were also calculated.

### 2.6. Statistics and Data Presentation

Continuous data are presented as mean ± standard deviation.

The study population was divided into approximate quartiles according to the age of the subjects. In each of these age strata, heart rates, and the slopes of the HRV/heart rate (or HRV/averaged RR) regressions and their regression residuals were compared between sexes using two-sample, two-tailed *t*-tests assuming different variances of the compared values. Separately for each sex, differences between the age quartiles were tested using one-way ANOVAs with Tukey’s post hoc tests. The regression residuals between the HRV/heart rate and HRV/averaged RR linear regressions were compared by paired two-tailed *t*-tests.

The slopes of the HRV/heart rate (or HRV/averaged RR) regressions were also related to age using the data from individual subjects. The relationships of the slopes to age were displayed together with curvilinear regression analyses. Spearman correlation coefficients were used to test the statistical significance of these relationships.

The relationship between the intra-subject HRV/heart rate regression slope and age might be influenced by the confidence intervals of the regression slopes, which might also be age dependent. Therefore, the cases which showed statistical significance for the dependence of the intra-subject slopes on age were also analysed for the age-dependence of the upper and lower 95% single-sided intra-subject confidence intervals of the slopes to ascertain that the observations were not biased because of the imprecisions of the intra-subject regression analyses.

ECG data processing was performed by in-house-developed software routines programmed in C/C++ (Microsoft Visual Studio 2022, version 17.4.3, Redmond, WA, USA). Statistical evaluations were performed using the IBM SPSS Statistics package (version 29.0.0.0, Armonk, NY, USA). *p*-values were considered to be statistically significant if below 0.05. *p*-values above this level are not presented but the acronym NS is shown to signify no statistical significance. Because of the interdependency of the compared data, no significance adjustment for multiplicity of test was performed. All statistical tests performed are reported.

## 3. Results

### 3.1. Population

While every child or adolescent who agreed to participate was investigated, the data used in the analysis presented here excluded those who were on drugs potentially affecting cardiac electrophysiology, or on hormonal contraceptives, those with cardiac abnormalities, and participants undergoing sex transversal procedures.

The investigation was performed in 1094 participants. Of these, 47 subjects (4.5%) were subsequently excluded for the previously listed reasons. Of those remaining, valid heart rate and HRV data for at least six postural positions were available from 1016 subjects who all had normal physical investigations at enrolment. The others either did not complete at least six of the seven postural positions because of physical discomfort, pre-syncopal symptoms, or nausea or occasional vomiting in small children, or had more than one of the postural positions excluded because of ectopic beats in all possible 5 min sub-sections.

**Figure 1:**
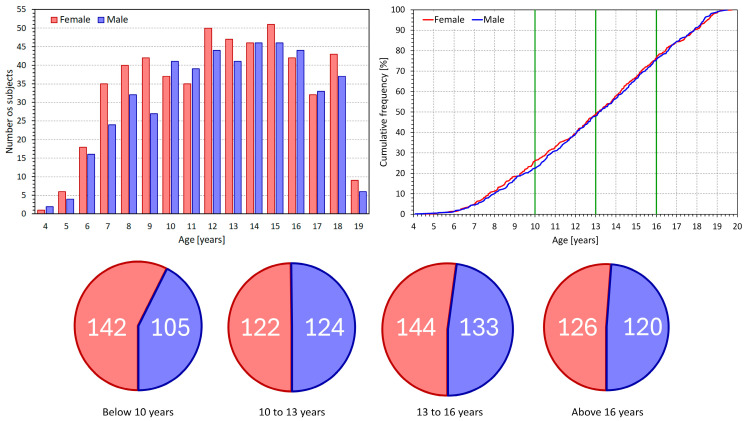
The figure describes the composition of the study population. The **upper left panel** shows the numbers of subjects of different years of age (data truncated to the full year, e.g., a subject aged 10 years and 11 months is shown in the 10-year age group). The **upper right panel** shows the cumulative age distribution. The vertical green lines show the approximation of the age-related quartiles of the population. The composition of the quartiles is shown in the pie charts in the bottom row of the Figure (note that the sizes of the pie charts are made proportional to the sizes of the quartiles—e.g., the 13 to 16 years chart is marginally larger than the others—showing that the departures from exact quartiles were of no importance). In all panels, the red and blue bars, lines, and slices correspond to female and male subjects, respectively. The numbers in the pie charts show the exact numbers of subjects.

These 1016 subjects formed the population of the investigation presented in this text. Of these, 534 were females (aged 12.9 ± 3.7 years) and 482 were males (aged 13.1 ± 3.6 years). Data for all seven postural positions were available from 947 subjects (93.2%). The composition of the population is shown in Figure 1. The distribution of ages shows that dichotomies of 10, 13, and 16 years reasonably approximated the borders between age-defined quartiles of the population (see the pie-charts at the bottom of Figure 1).

### 3.2. Heart Rate and Heart Rate Variability Indices

**Figure 2:**
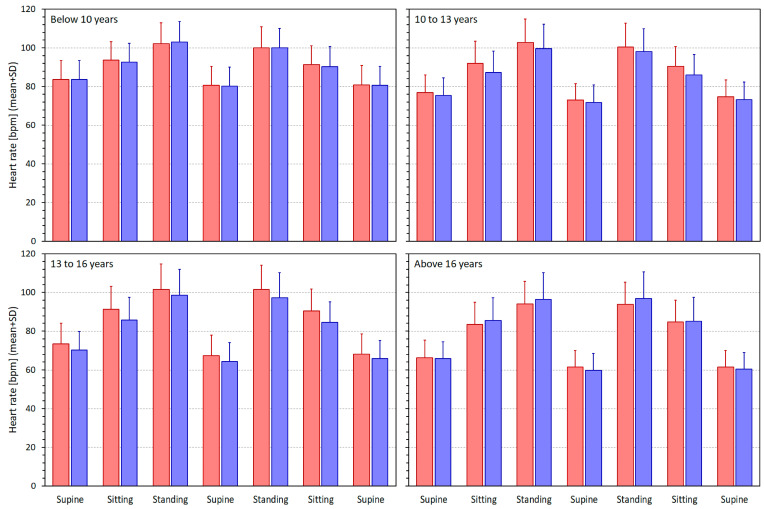
Heart rates measured in different quartiles of the population (see the top left corners of the panels). The postural positions during which the measurements were made are shown below the panels in the order they were conducted during the experiments. The red and blue bars correspond to females and males, respectively. bpm—beats per minute; SD—standard deviation.

Figure 2 shows the heart rates measured in different postural positions in age-related quartiles of the population.

The ranges of heart rates (i.e., the ranges between subject-specific minima and maxima over which the intra-subject linear regressions of HRV indices were calculated) were, in the group below 10 years of age, 24.7 ± 8.7 and 25.3 ± 8.0 bpm in females and males, respectively (NS between females and males). The corresponding values in the groups 10 to 13 years, 13 to 16 years, and above 16 years were 32.0 ± 10.5 and 30.6 ± 10.1 bpm (NS), 37.6 ± 10.4 and 36.9 ± 10.9 bpm (NS), and 35.9 ± 9.9 and 40.0 ± 12.4 bpm (*p* = 0.0044), respectively.

When the heart rate measurements were expressed as the RR interval averages, the following intra-subject max—min ranges of averaged RR intervals were found in females and males of the population quartile groups: 187 ± 73 and 192 ± 70 ms (NS), 261 ± 82 and 263 ± 88 ms (NS), 340 ± 112 and 355 ± 111 ms (NS), and 383 ± 115 and 421 ± 127 ms (*p* = 0.0154). With both heart rate and averaged RR data, the differences between these max—min ranges were highly statistically significant between the age-related quartiles (*p* < 0.0001 for both sexes).

**Figure 3:**
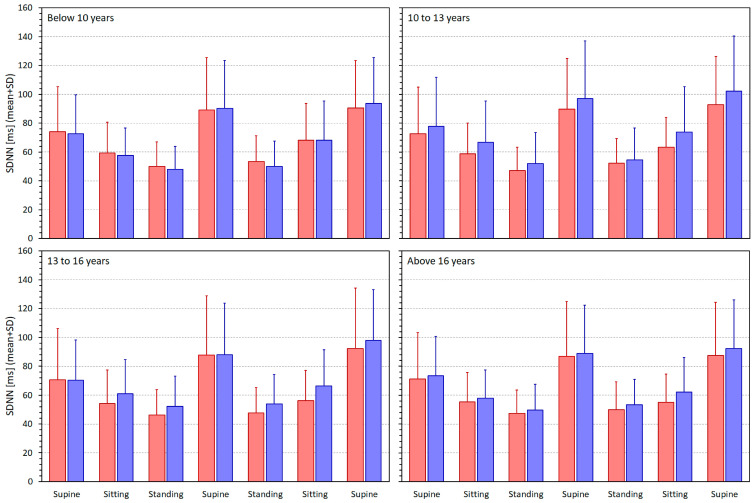
The SDNN (standard deviation of normal-to-normal RR intervals) values measured in different quartiles of the population. The layout of the figure, including the colour distinction, corresponds to the layout of Figure 2.

**Figure 4:**
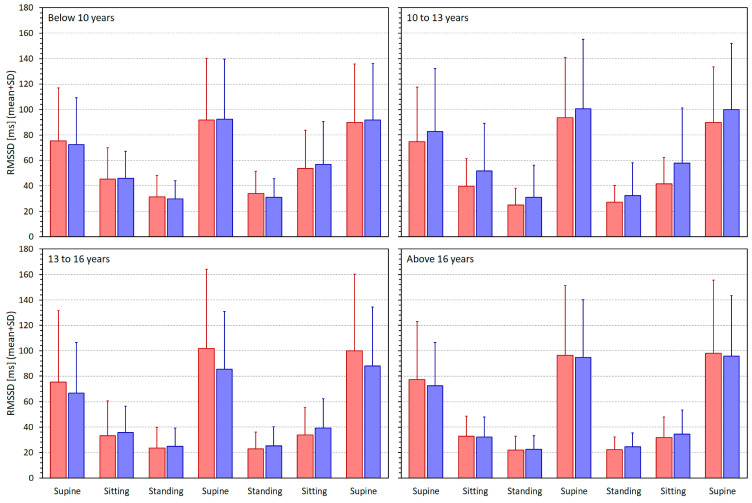
The RMSSD (root mean square of successive differences between normal-to-normal RR intervals) values measured in different quartiles of the population. The layout of the figure, including the colour distinction, corresponds to the layout of Figure 2.

**Figure 5:**
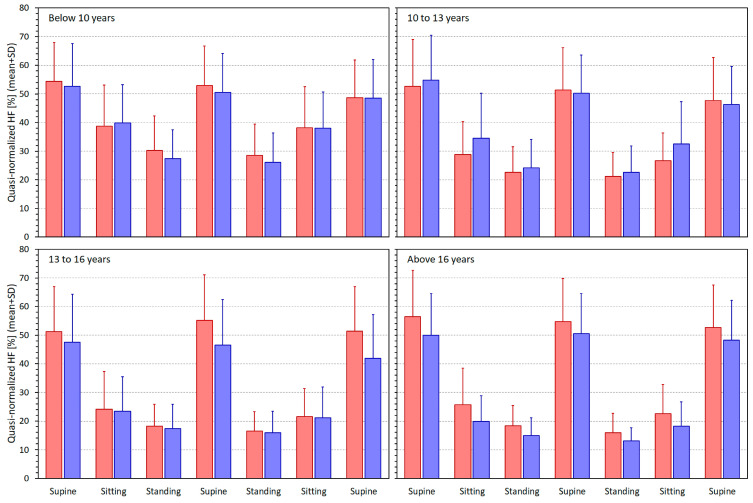
Quasi-normalised high-frequency spectral heart rate variability components (qnHF—see the text for details) measured in different quartiles of the population. The layout of the figure, including the colour distinction, corresponds to the layout of Figure 2.

**Figure 6:**
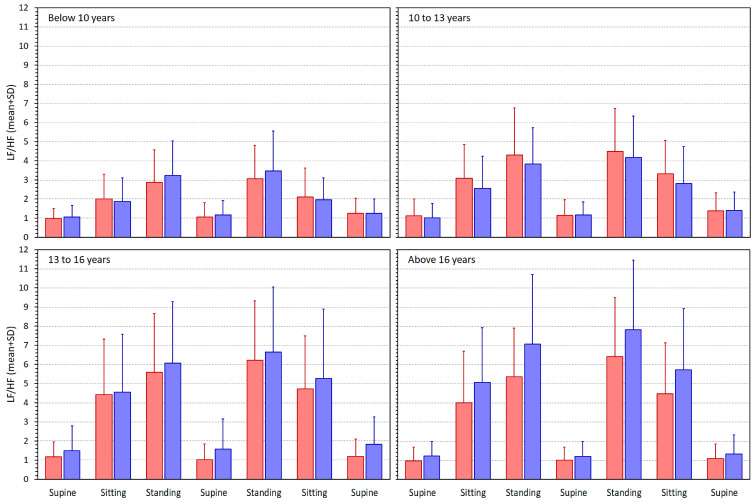
The proportions of low- and high-frequency spectral heart rate variability components (LF/HF—see the text for details) measured in different quartiles of the population. The layout of the figure, including the colour distinction, corresponds to the layout of Figure 2.

Figure 3, Figure 4, Figure 5 and Figure 6 show similar displays of measured SDNN, RMSSD, qnHF, and LF/HF indices.

With the SDNN measurements, the intra-subject linear regressions relating the values to the averaged RR intervals led systematically to lower regression residuals in comparison to the linear regression relating the values to heart rates. The differences in the regression residuals were all statistically significant, with *p*-values ranging from <0.0001 to 0.0225, with the exception of males between the ages 10 and 13 years, where the comparison of the regression residuals showed the same direction of the differences but without statistical significance. In younger children, that is the groups below 10 years and between 10 and 13 years, the regression residuals were significantly smaller in females compared to males (*p* = 0.0086 and 0.0315), but in the older children and adolescents the residuals showed no statistically significant sex differences.

The same comparisons with preference to intra-subject RMSSD/averaged RR regressions were observed with the RMSSD/heart rate regressions, leading to higher residuals in all population sub-groups (*p* < 0.0001 for both sexes in all population quartiles). The sex comparison of the RMSSD/averaged RR regression residuals showed no statistical significance in any population quartiles.

Numerically, preference for the qnHF/averaged RR regression was also observed but while the comparison with the qnHF/heart rate regression residuals was statistically significant in the complete population (*p* < 0.0001), a statistically non-significant trend in the same direction was seen in several of the population sub-groups (without any obvious sex or age relationship). In the complete population, there was no sex difference in the qnHF/averaged RR regression residuals.

In the study population, there was no statistically significant difference between the residuals of the (LF/HF)/heart rate and (LF/HF)/averaged RR regressions. Only a trend towards lower (LF/HF)/heart rate regressions residuals was noted. The differences in population sub-groups were inconsistent. Therefore, to make the investigations comparable between different HRV indices, the (LF/HF)/averaged RR regressions were used.

### 3.3. HRV/RR Relationship in the Complete Population

**Figure 7:**
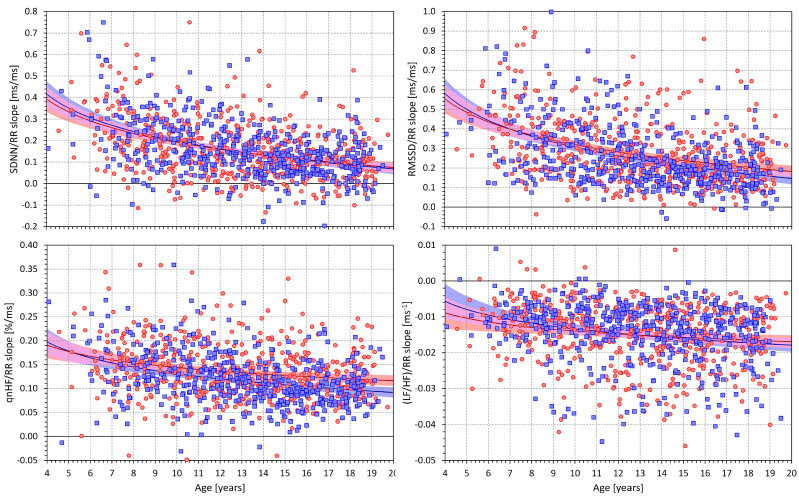
Scatter diagrams of the age of the subjects and intra-subject slopes of the linear regressions of the SDNN/averaged RR interval (**upper left panel**), RMSSD/averaged RR interval (**upper right panel**), qnHF/averaged RR interval (**lower left panel**), and (LF/HF)/averaged RR interval (**lower right panel**). In each panel, the red circles and blue squares correspond to female and male subjects, respectively, the red and blue bold lines are curvilinear regressions through the displayed data of female and male subjects, respectively, the light red and light blue areas are the 95% confidence bands of the displayed regression lines, and the light violet areas are the overlaps between the confidence bands of both sexes. SDNN—standard deviation of normal-to-normal RR intervals, RMSSD—root mean square of successive differences between normal-to-normal RR intervals, qnHF—quasi-normalised high-frequency spectral heart rate variability components, and LF/HF—proportion between low- and high-frequency spectral heart rate variability components.

Figure 7 shows scatter diagrams depicting the age and intra-subject HRV/averaged RR regression slopes for all the considered HRV indices. The scatter diagrams are shown together with sex-specific log/linear population regressions. Overall, the scatter diagrams correspond to what has already been observed in the comparison of Figure 2 with Figure 3, Figure 4, Figure 5 and Figure 6. When heart rate increases (i.e., the averaged RR interval decreases), the SDNN, RMSSD, and qnHF indices decrease, while, in contrast, the LF/HF proportions increase.

More importantly, the scatter diagrams in Figure 7 also show that the intra-subject HRV/averaged RR regression slopes depend on age. The Spearman correlation coefficients between the SDNN/RR slopes and age were −0.374 and −0.408 for females and males, respectively (both *p* < 0.0001). Similar findings were obtained for correlations between the RMSSD/RR slopes and age, with correlation coefficients of −0.350 and −0.408 (both *p* < 0.0001). While still highly statistically significant, the corresponding slope vs. age correlation coefficients were lower for qnHF (−0.231 and −0.351, both *p* < 0.0001) and even lower for LF/HF proportion (−0.163 and −0.244, both *p* < 0.0001).

**Figure 8:**
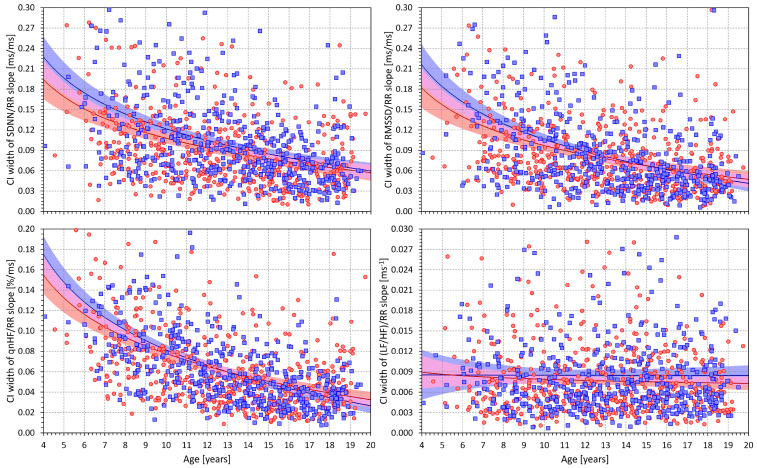
Scatter diagrams of the age of the subjects and intra-subject widths of the dual-sided 90% confidence intervals (CI) of slopes of the linear regressions of the SDNN/averaged RR interval (**upper left panel**), RMSSD/averaged RR interval (**upper right panel**), qnHF/averaged RR interval (**lower left panel**), and (LF/HF)/averaged RR interval (**lower right panel**). The layout of the figure and the meaning of the symbols, lines, and coloured areas are the same as in Figure 7.

Figure 8 shows that the widths of the confidence intervals of intra-individual HRV/RR slopes were also dependent on age for all HRV indices apart from the LF/HF proportion. The Spearman correlation coefficients between the age and the widths of the slope confidence intervals for females and males were −0.395 and −0.407 for the SDNN (both *p* < 0.0001), −0.384 and −0.435 for RMSSD (both *p* < 0.0001), −0.464 and −0.587 for qnHF (both *p* < 0.0001), and −0.021 and −0.006 for LF/HF (both NS), respectively.

For the SDNN, RMSSD, and qnHF, the width of the slope confidence intervals thus needs to be considered when studying the relationship to age. When incorporating this aspect and studying the low 95% confidence intervals of the intra-subject HRV/RR slopes, significant relationships to age were still found for the SDNN (Spearman correlation coefficients of −0.223 and −0.223 for females and males, both *p* < 0.0001) and for the RMSSD (coefficients of −0.255 and −0.298 for females and males, both *p* < 0.0001).

For the qnHF, however, the statistical significance of the slope of relationship to age became non-significant when studying the low 95% confidence intervals of the qnHF/RR slopes (Spearman correlation coefficients of +0.070 and +0.020 for females and males, both NS).

### 3.4. HRV/RR Relationship in Population Quartiles

**Figure 9:**
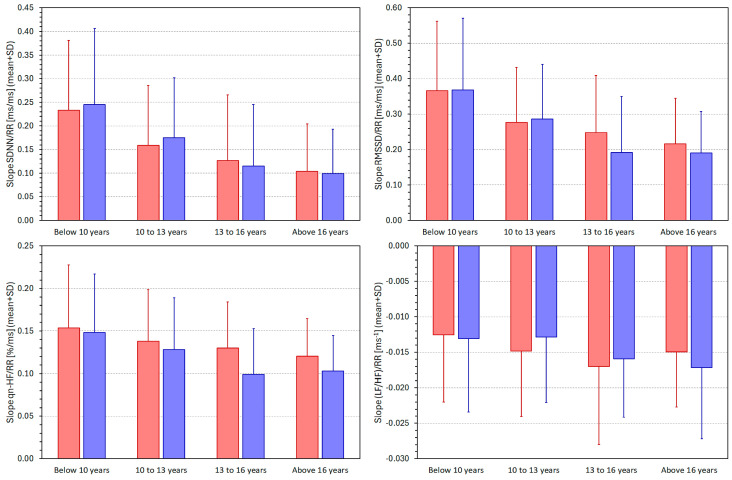
Comparisons between the intra-subject slopes of the linear regressions of the SDNN/averaged RR interval (**upper left panel**), RMSSD/averaged RR interval (**upper right panel**), qnHF/averaged RR interval (**lower left panel**), and (LF/HF)/averaged RR interval (**lower right panel**) in different age-defined quartiles of the population. The population quartiles are shown below each panel. The red and blue bars correspond to females and males, respectively. SDNN—standard deviation of normal-to-normal RR intervals, RMSSD—root mean square of successive differences between normal-to-normal RR intervals, qnHF—quasi-normalised high-frequency spectral heart rate variability components, LF/HF—proportion between low- and high-frequency spectral heart rate variability components, and SD—standard deviation.

Figure 9 summarises the individual HRV/average RR regression slopes in the different quartiles of the population. The observed trends correspond to the graphical images seen in Figure 7. With advancing age, the slopes of the intra-subject HRV/average RR regressions decrease. That means that for the SDNN, RMSSD, and qnHF, the values of the indices are becoming less dependent on the underlying heart rate while the opposite is seen for the LF/HF proportion (note that the slopes of the (LF/HF)/RR regressions are negative). The differences between the population quartiles were statistically significant for all HRV indices and both sexes (*p* < 0.0001 in all cases, with the exception of the (LF/HF)/RR slopes, where the significances were *p* = 0.001 and *p* = 0.0006 for females and males, respectively).

The bar graphs in the panels of Figure 9 also show that we have seen few differences between female and male subjects. Significant sex differences were only noted in the 13 to 16 years age group for the RMSSD (*p* = 0.001) and qnHF (*p* < 0.0001) indices.

**Figure 10:**
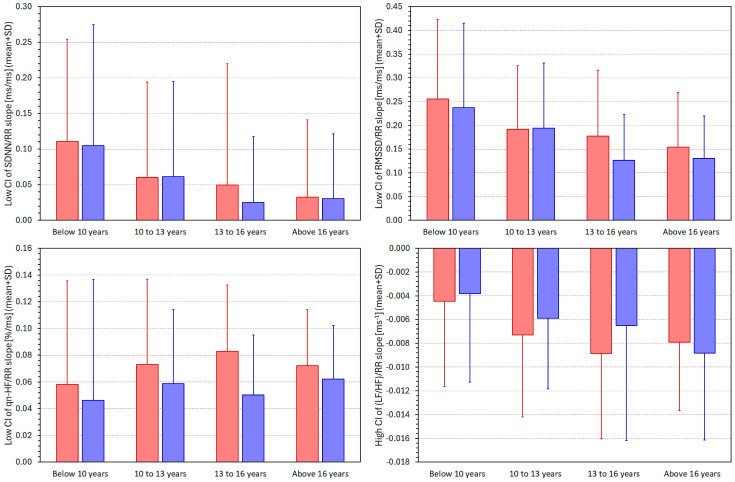
Comparisons between the lower, single-sided 95% confidence intervals (CI) of the intra-subject slopes of the linear regressions of the SDNN/averaged RR interval **(upper left panel**), RMSSD/averaged RR interval (**upper right panel**), and qnHF/averaged RR interval (**lower left panel**), and the upper single-sided 95% confidence intervals (CI) of the intra-subject slopes of the linear regressions of the (LF/HF)/averaged RR interval (**lower right panel**). The comparisons are shown for different age-defined quartiles of the population. The layout of the figure and the colour coding correspond to that of Figure 9.

Figure 10 shows the same comparisons as presented in Figure 9 but computed for the low 95% confidence intervals of the SDNN/RR, RMSSD/RR, and qnHF/RR slopes, and for the high 95% confidence intervals of the (LF/HF)/RR slopes. Statistical analyses of these data confirmed the observation already presented in Figure 8. Irrespective of the age-related differences in the precision of the HRV/RR slope calculations, the slopes of the SDNN/RR and RMSSD/RR regressions were statistically significantly decreasing with increasing age (*p* < 0.0001 for both cases and both sexes). The low 95% confidence intervals of the qnHF/RR regressions differed significantly between the population quartiles only for females (*p* = 0.0070) but not for males. The 95% confidence intervals of the (LF/HF)/RR regressions are shown only for completeness since, as already shown, the width of the confidence intervals was not found to be age dependent.

## 4. Discussion

The study leads to somewhat unexpected observations. Based on purely theoretical considerations, we expected that if any age-related differences in the HRV/heart rate relationship are found, the slopes would be shallower in younger children because of the shorter spans between the minimum and maximum heart rates triggered by postural manoeuvring. The observation of the exact opposite relationship to age (as far as the SDNN, RMSSD, and qnHF indices are concerned) has implications for the understanding of the development of heart rate control in children. In this sense, several aspects need to be considered.

There is an important technical difference between the HRV indices that we investigated. The SDNN and RMSSD are “global” variability HRV parameters for which reduction at higher heart rates might be expected, among others, for purely technical reasons [40], since even the same level of, say, respiratory arrhythmia leads to larger absolute values of RR variability when influencing longer rather than shorter RR intervals. On the contrary, the qnHF and LF/HF indices are less influenced by such technical factors since the technical influences of the LF and HF components practically cancel each other in the calculation of these indices. In any case, these technical reasons can hardly explain the differences between younger and older children.

Considering the spectral indices, the main observed differences between the age groups were the steeper qnHF and shallower LF/HF relationships to heart rate in younger children. These need to be interpreted based on the physiological background of these two HRV indices. The LF components of HRV represent a mixture of sympathetic and vagal modulations and the HF components are associated with vagal influences on RR periodicity [1]. Nevertheless, direct comparison of LF and HF components measured in different recordings might be problematic [32,34]. For that reason, the LF/HF ratios and normalised LF and HF components have been introduced [1]. As already explained, in this study, we approximated normalised HF components by their quasi-normalised variants [49]. As mentioned in the Methods section, increases in LF/HF ratio might be interpreted as a surge in sympathetic modulations, and increases in qnHF components indicate augmentation of vagal effects. Data shown in Figure 2 and Figure 5 show that in comparison to older age groups, protocol-induced qnHF changes are smaller in younger children but that this is offset by lesser heart rate changes. Thus, the steeper qnHF/RR slopes in younger children reflect more abrupt qnHF changes over a narrower window of RR averages. The situation with LF/HF changes is the opposite. The protocol-induced LF/HF changes in younger children are, in comparison to older children, less pronounced compared to the changes in RR averages.

Thus, our observations agree with the previous suggestions that vagal reactions to physical activity are well-developed in small children [51,52,53] but are at odds with the proposal that, in children, the sympathetic influence on heart rate shows a linear reduction with age [54]. We also have not observed a gradual age-related increase in the vagal cardiac influence [54]. More likely, our observations lead to the suggestion that while both sympathetic and vagal influences on heart rate co-exist in younger children, the sympatho-vagal balance develops progressively and allows vagal control to suppress the sympathetic drive towards higher heart rates only gradually with advancing age during puberty and early adolescence.

As already stated, our approach to the data analysis presented in this text is very different compared to the previous suggestions of correcting the HRV values for the underlying heart rate [37,39,40,41] (previous suggestions might perhaps be seen in a way similar to the correction of the measured QT interval duration to the corresponding heart rate, or the correction of atmospheric pressure to altitude). Postural changes activate different autonomic reactions and reflexes [55,56] which, among other variables, affects heart rate and HRV [34]. Therefore, HRV does not respond to heart rate, but both phenomena react—possibly differently—to the same broad control mechanisms. Since our analyses grouped data obtained under different conditions of autonomic control, it would be very inappropriate to assume that a “corrected” HRV value might be obtained that would be informative regarding autonomic status under such variable circumstances.

This does not mean that it would not be legitimate to ask whether a physiologically relevant HRV/HR relationship exists in a well-defined population recorded under strictly the same conditions of cardiovascular autonomic balance. While a principally different analysis of our data might answer the question of whether such a population-based HRV/HR relationship differs between sexes, different groups, and different postural positions, the analysis that we present in this text addresses very different aspects of autonomic heart rate and HRV control.

### Limitations

Our study and the presented analyses included simplifications that lead to both physiological and technical limitations. Since the aim of the study was to contribute to the understanding of how autonomic heart rate modulations develop in children and adolescents, we have not considered HRV indices that might be difficult to interpret in this sense. Further studies are needed to investigate the age-related development of other HRV indices such as symbolic dynamics of RR intervals [24,57], HRV entropy estimates [58], and fractal properties of RR tachograms [59] and their detrended fluctuation analysis [60], and other techniques. For organisational and limited support reasons, we were unable to collect continuous beat-to-beat blood pressure recordings and were thus unable to support our analyses by non-invasive assessment of baroreflex sensitivity [61,62]. For similar reasons, we were also unable to record respiration. Attempts were made to approximate respiration frequencies from ECG morphology using the algorithms validated in supine adults [63]. Nevertheless, these attempts were not successful, especially in the sitting and standing positions. Even in the unlikely scenario [64] that the respiration frequency in the very young children exceeded 24 cycles per minute, and thus contributed to the RR interval variation outside the spectral band of 0.15–0.40 Hz, the vagal heart rate modulations would have been underestimated; i.e., our conclusion on the absence of a gradual increase in vagal cardiac influence during childhood would not be compromised. While the demographic and clinical status data that we collected also included the time since last menstruation in post-puberty females, we have not used this information in the present analyses [65]. The catchment area of our study included regions with very few minorities among the population. The study was consequently conducted mostly in Caucasians, and we are unable to present any meaningful race sub-studies [66,67]. To facilitate problem-free enrolment in the study, no data on school reports were collected, although differences in cognitive function might have some influence on stress due to the study protocol. Finally, the presented analyses included only healthy children. We are thus unable to comment on the possible consequences of dysautonomia and general neuropathies.

## 5. Conclusions

Despite these limitations, our study suggests that sympathetic and vagal influences on heart rate are present in younger children. The study supports neither the gradual decrease in sympathetic influence nor the gradual increase in vagal cardiac influence during childhood and adolescence. Rather, the sympatho-vagal balance gradually develops and allows vagal control to suppress the sympathetic drive towards higher heart rates, leading to the observed heart rate differences between the early childhood and adolescent years.

## Data Availability

The data supporting the conclusions of this article will be made available by the authors pending approval by the Ethics Committee of University Hospital Brno and by the Steering Committee of the main project of paediatric data collection. Requests for the data are to be sent to the corresponding author together with a detailed plan of the proposed analyses.
